# Cyclotorsion as a structural and functional biomarker of ocular and neuroophthalmic diseases

**DOI:** 10.3389/abp.2026.17270

**Published:** 2026-09-18

**Authors:** Agata Joanna Ordon, Piotr Jerzy Loba

**Affiliations:** Department of Binocular Vision Pathophysiology and Strabismus, University Barlicki Hospital No. 1, Medical University of Lodz, Lodz, Poland

**Keywords:** cyclorotation, cyclotorsion, eye movements, ocular motility, strabismus

## Abstract

Cyclotorsion, defined as rotation of the eye around its anteroposterior axis, is an important but often underrecognized component of ocular motility. This review summarizes the physiological basis of cyclotorsion, methods of subjective and objective assessment, and the clinical relevance of torsional deviations in ocular and neuro-ophthalmic disorders. Particular emphasis is placed on objective measurement using the disc–foveal angle and its role in diagnosing and monitoring conditions such as trochlear nerve palsy, skew deviation, pulley disorders, inferior oblique overaction, thyroid eye disease, heavy eye syndrome, and sagging eye syndrome. Quantitative assessment of cyclotorsion may improve diagnostic accuracy, support surgical planning, and provide an objective marker of treatment outcome.

## Introduction

Cyclotorsion, also referred to as torsional eye movement, is the rotation of the eyeball around its anteroposterior (visual) axis. The eye rotates around a central point of rotation within a three-dimensional coordinate system, in which horizontal, vertical, and torsional rotations occur around orthogonal axes, with Listing’s plane serving as an important reference for describing ocular rotations from the primary position, as illustrated in Figure 1. It represents an essential element of ocular motility, supporting accurate eye alignment, stabilization of retinal image orientation, and maintenance of binocular vision. It may describe either the torsional position of a single eye, known as cyclodeviation (incyclotorsion or excyclotorsion), or coordinated torsional movements of both eyes (Brodsky, 2022; Maxwell and Schor, 2006). Torsional movements of both eyes include cycloversion, a conjugate movement in the same direction, and cyclovergence, a disjunctive movement in opposite directions, whereas cycloduction refers to the torsional movement of a single eye. In the absence of cyclotorsion, the vertical meridian passing through the center of the cornea remains aligned with the true vertical. Incyclotorsion occurs when the superior pole of the eye rotates nasally, resulting in the optic disc being positioned lower relative to the fovea, while excyclotorsion involves temporal rotation of the superior pole, causing the optic disc to lie higher than the fovea (Spielmann, 2002). These torsional adjustments are essential for maintaining stable visual perception and appropriate spatial orientation, particularly during head and body movements.

**FIGURE 1:**
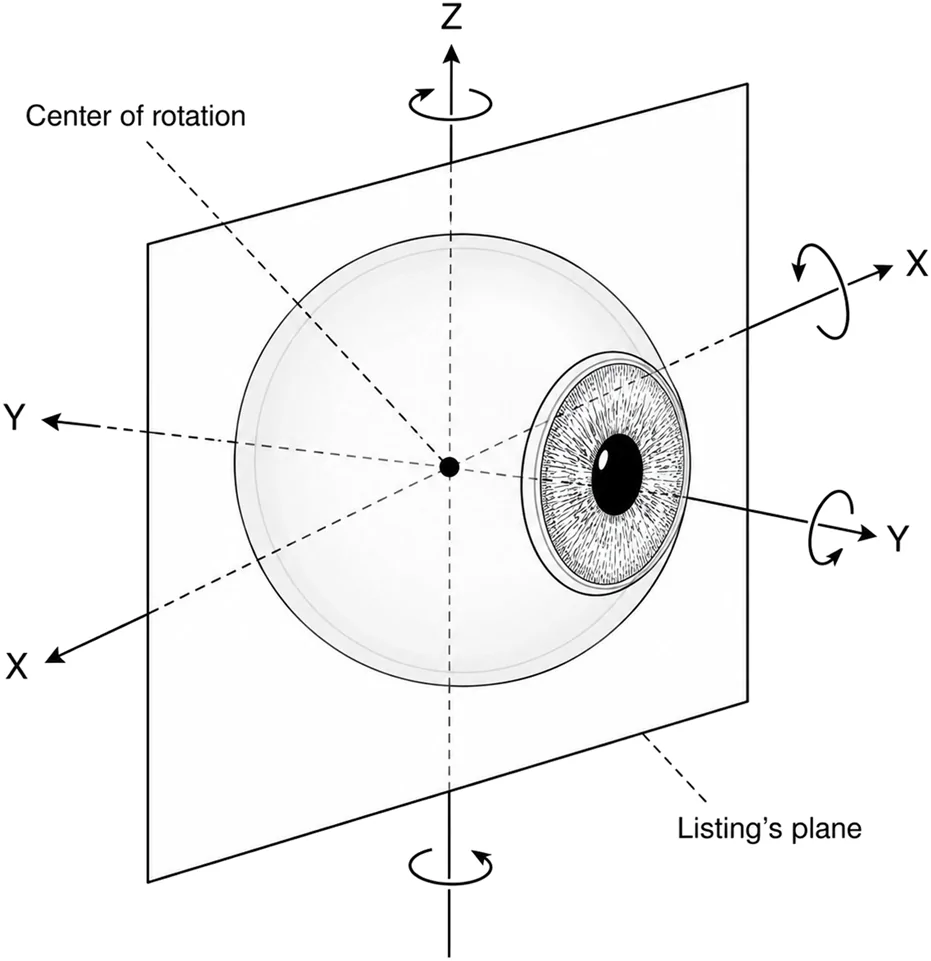
Schematic representation of ocular rotation axes and Listing’s plane.

Beyond its role in ocular motility, cyclotorsion has emerged as a valuable structural and functional biomarker in a wide range of ocular and neuro-ophthalmic disorders (Brodsky, 2022; Lemos and Eggenberger, 2013). Abnormal torsional alignment may reflect dysfunction of the extraocular muscles, cranial nerves, vestibular pathways, or central nervous system structures involved in gaze control. Quantitative assessment of cyclotorsion can provide important diagnostic information in conditions such as superior oblique palsy, skew deviation, thyroid eye disease, vestibular disorders, and various neuro-ophthalmic syndromes (Brodsky, 2022; Lemos and Eggenberger, 2013; Zurevinsky, 2022; Brodsky et al., 2006). Moreover, structural indicators of cyclotorsion, including changes in the disc-foveal angle and fundus torsion, may correlate with functional outcomes and disease severity. Advances in ocular imaging and eye-tracking technologies have enabled increasingly precise and objective measurement of cyclotorsion, facilitating its integration into both clinical practice and research. As a result, cyclotorsion is gaining recognition not only as a manifestation of ocular motor dysfunction but also as a potential biomarker for the diagnosis, monitoring, and evaluation of treatment outcomes in ocular and neuro-ophthalmic disease. Unlike previous reviews focusing on selected aspects of ocular torsion, this review integrates its physiological basis, assessment methods, and clinical relevance across ocular and neuro-ophthalmic disorders. It also highlights the emerging role of cyclotorsion as a quantitative structural and functional biomarker. Relevant literature was identified through targeted searches in PubMed and reference lists of key publications, with emphasis on studies addressing the physiology, measurement, and clinical relevance of cyclotorsion. The clinical conditions discussed were selected to represent the main neurogenic, vestibular, restrictive, and mechanical mechanisms in which cyclotorsion has diagnostic or therapeutic relevance.

## Physiological mechanisms of ocular cyclotorsion

Physiological ocular cyclotorsion is a finely regulated component of normal binocular motor control that serves to maintain stable retinal image orientation during changes in head and body position. The principal physiological mechanism underlying ocular cyclotorsion is the ocular counter-roll (OCR), a vestibulo-ocular reflex generated by activation of the otolith gravity sensors in the inner ear during static head tilt (Reschke et al., 2018). OCR partially compensates for head rotation in the roll plane, thereby contributing to the stabilization of gaze, retinal image orientation, and postural control. This response is mediated by the otolith organs, particularly the utricles, which detect gravitational acceleration and transmit signals via the vestibular nuclei to the oculomotor and trochlear nuclei. Torsional eye movements are generated primarily by the coordinated actions of the superior and inferior oblique muscles, assisted by the vertical rectus muscles, whose torsional actions depend on the position of gaze (Horn and Leigh, 2011; Demer, 2003; Kushner, 1986). In humans, OCR compensates for approximately 10%–25% of head tilt, reflecting a balance between retinal image stabilization and maintenance of binocular alignment (Kheradmand and Winnick, 2017). In addition to vestibular input, ocular cyclotorsion is modulated by visual and central neural mechanisms that integrate sensory information to optimize ocular motor performance. The cerebellum plays a key role in calibrating vestibulo-ocular responses and maintaining binocular alignment through adaptive control of eye movements, while brainstem pathways coordinate the vestibulo-ocular reflex and torsional gaze stabilization. These physiological torsional adjustments preserve retinal image stability, facilitate binocular fusion, and contribute to accurate perception of spatial orientation during changes in head and body position (Brodsky, 2022; Kheradmand and Zee, 2011). A brief summary of the physiological mechanisms underlying ocular cyclotorsion and the ocular counter-roll reflex is presented in Figure 2.

**FIGURE 2:**
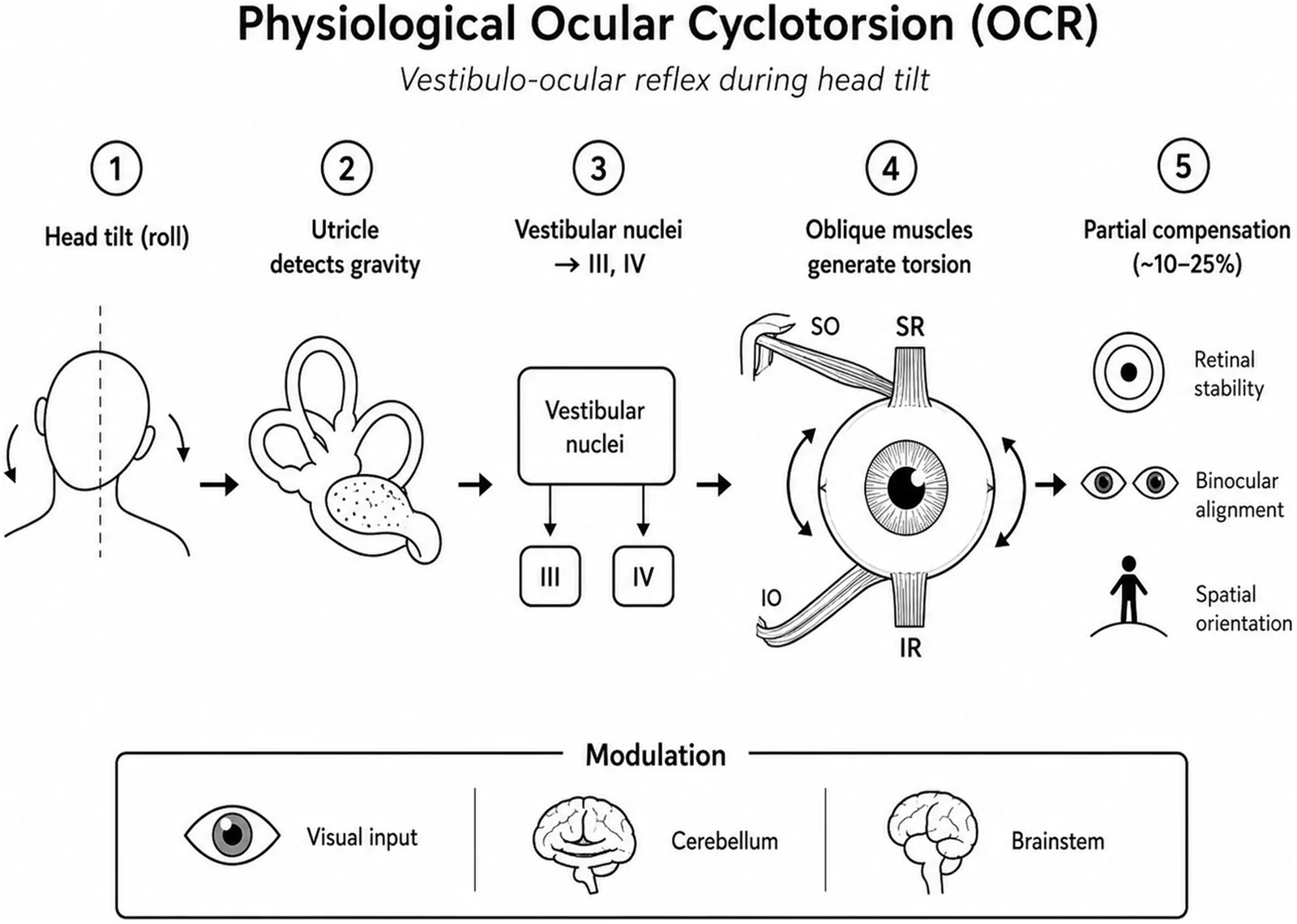
Physiological ocular cyclotorsion mediated by the ocular counter-roll reflex.

## Extraocular muscles responsible for cyclotorsion

The extraocular muscles play a crucial role in the precise control of eye movements, including torsional (cyclorotational) movements. Incyclotorsion, defined as the inward rotation of the eye around its anteroposterior axis, and excyclotorsion, defined as outward rotation, are primarily mediated by the oblique muscles. The superior oblique muscle is the principal muscle responsible for incyclotorsion, whereas the inferior oblique muscle serves as the primary excyclotortor. These actions are supplemented by the vertical rectus muscles: the superior rectus muscle contributes secondarily to incyclotorsion, while the inferior rectus muscle contributes secondarily to excyclotorsion (Rucker and Tomsak, 2005; Horn and Leigh, 2011). Although torsional movements represent secondary actions of the rectus muscles, they are important for maintaining ocular alignment and visual stability during eye and head movements. The coordinated activity of these muscles enables precise torsional adjustments that support binocular vision, image stabilization, and spatial orientation. Disturbances in the balance of torsional muscle actions may result in abnormal cyclotorsion and are commonly observed in various ocular motility and neuro-ophthalmic disorders. A summary of the primary, secondary, and tertiary actions of the extraocular muscles is presented in Table 1.

**TABLE 1:** Primary, secondary, and tertiary functions of the extraocular muscles.

Extraocular muscle	Primary function	Secondary function	Tertiary function
Superior oblique	Intorsion	Depression	Abduction
Inferior oblique	Extorsion	Elevation	Abduction
Superior rectus	Elevation	Intorsion	Adduction
Inferior rectus	Depression	Extorsion	Adduction
Medial rectus	Adduction	-	-
Lateral rectus	Abduction	-	-
Levator palpebrae superioris	Upper eyelid elevation	-	-

## Imaging and quantitative assessment of cyclotorsion

Cyclotorsion may be assessed using both subjective and objective methods. Subjective evaluation reflects the patient’s perceptual experience of torsion and is commonly performed using the synoptophore, double Maddox rod test, or Harms tangent screen (Llorente-La Orden et al., 2022; Klainguti et al., 1992; Flodin et al., 2020). The synoptophore is useful for assessing cyclofusion and estimating the amount of torsional deviation that may need to be corrected surgically to relieve symptoms. The double Maddox rod test is widely used to quantify perceived torsion by asking the patient to rotate arrays of parallel cylindrical lenses until the perceived lines appear parallel; however, the use of red and white filters may influence torsional perception, particularly in the eye viewing through the red filter. Therefore, although this test is useful for estimating overall cyclodeviation, it may be less reliable for identifying the eye primarily responsible for the torsional abnormality (Simons et al., 1994). The Harms tangent screen allows quantitative assessment of torsion in different gaze positions and is particularly valuable in the preoperative evaluation of patients with trochlear nerve palsy.

In routine ophthalmological practice, assessment of cyclotorsion is often based mainly on subjective testing and patient-reported symptoms. However, patients rarely report image tilting spontaneously, and the clinical expression of cyclotropia is highly variable. In some cases, torsional diplopia can be elicited only under strongly dissociative testing conditions, whereas in others, marked cyclotropia may remain asymptomatic because of adaptive mechanisms such as cyclofusion, suppression, or the use of spatial orientation cues. As a result, subjective tests may yield normal findings despite clear objective evidence of torsional deviation on fundus examination. Spontaneously reported torsional diplopia usually suggests a relatively recent onset of strabismus, whereas longstanding or congenital torsional deviations may produce intermittent symptoms or remain entirely asymptomatic. Thus, even pronounced fundus torsion may be associated with a broad clinical spectrum, ranging from constant disturbing tilted diplopia to occasional diplopia or complete absence of symptoms.

Objective cyclotorsion refers to measurable rotation of the globe around its visual axis and is assessed independently of the patient’s subjective perception (Kushner and Hariharan, 2009). It is commonly quantified using anatomical landmarks on fundus imaging, particularly the relationship between the optic disc and the fovea. The disc–foveal angle (DFA), also referred to as the foveopapillary angle (FPA), is defined as the angle between a horizontal reference line passing through the center of the optic disc and a line connecting the center of the optic disc with the fovea (Jonas et al., 2015; Choi et al., 2014). Changes in this angle reflect torsional rotation of the posterior pole, with increased values typically indicating excyclotorsion and reduced or negative values indicating incyclotorsion (Figure 3), depending on the measurement convention used.

**FIGURE 3:**
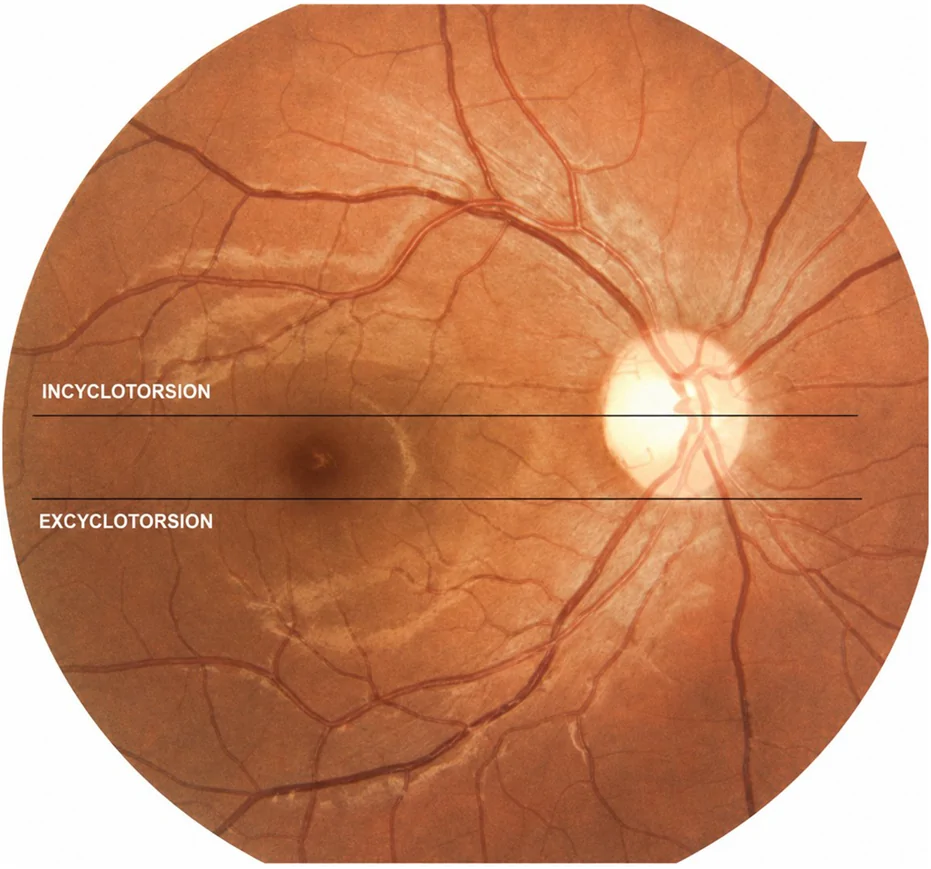
Representative fundus image illustrating the position of the optic disc relative to the fovea in ocular torsion. Incyclotorsion is associated with a lower position of the optic disc relative to the fovea, whereas excyclotorsion is associated with a higher position of the optic disc relative to the fovea.

Compared with subjective methods, objective assessment provides more reproducible and anatomically precise data, making it useful for diagnosing torsional misalignment, guiding surgical planning, and evaluating postoperative outcomes. DFA is widely regarded as the reference standard for assessing objective cyclotorsion. It is commonly measured using fundus photography, scanning laser ophthalmoscopy, or automated image-analysis software, with previous studies comparing these methods in terms of accuracy, reproducibility, and clinical applicability. (Jonas et al., 2015; Vélez Escolà et al., 2019). Comparison of subjective and objective methods for assessing ocular torsion is presented in Table 2.

**TABLE 2:** Comparison of subjective and objective methods for assessing ocular torsion.

Method	Subjective/objective	Main advantages	Main limitations	Reproducibility	Main use
Double Maddox rod	Subjective	Simple, fast, inexpensive; quantifies cyclodeviation	Requires cooperation; affected by dissociation and head position	Moderate	Routine assessment of subjective cyclotorsion
Synoptophore	Subjective	Measures torsional, horizontal and vertical deviation; assesses binocular function	Specialized equipment; time-consuming; requires cooperation	Moderate–good	Detailed strabismus assessment and follow-up
Harms tangent screen	Subjective	Assesses deviation in different gaze positions and incomitance	Operator-dependent; time-consuming; requires cooperation	Moderate	Complex strabismus and ocular motility disorders
Fundus photography/DFA	Objective	Quantitative, documentable measurement of fundus torsion	Requires good images; anatomical torsion may differ from subjective torsion	Good–excellent	Objective torsion measurement and follow-up
Scanning laser ophthalmoscopy (SLO)	Objective	High-resolution, precise and potentially dynamic measurement	Expensive; limited availability; fixation-dependent	Good–excellent	Research and complex torsion assessment
Digital image analysis	Objective	Automated, rapid, reproducible; reduces examiner bias	Depends on image quality and algorithm validation	Good–excellent	Objective monitoring, research and large datasets

Kanku et al. (2021), Jethani and Dave (2015), Lin et al. (2024) in 2021 our team developed Cyclocheck®, a web-based diagnostic tool for measuring objective cyclotorsion in both clinical and research settings (Simiera and Loba, 2017; Simiera et al., 2021). It is free to use, easily accessible, and offers a standardized, repeatable method to enhance measurement reliability. Analyzing anatomical landmarks on fundus photographs, such as the fovea center and optic disc edges, ensures precise and objective assessment. Its versatility makes it a valuable tool for everyday clinical practice and various research applications, supported by peer-reviewed validation. Compared with conventional manual fundus assessment, automated analysis may reduce examiner-dependent variability and improve efficiency, particularly when evaluating larger datasets or longitudinal changes. However, other objective techniques, including conventional fundus photography with manual or semi-automated analysis and scanning laser ophthalmoscopy, also allow reliable quantification of ocular torsion. Cyclocheck® should therefore be regarded as one of several available approaches rather than a reference standard. Its measurements remain dependent on image quality, correct identification of anatomical landmarks, and standardized image acquisition, and further validation in larger and independent populations would be valuable.

Although DFA is widely used as an objective measure of ocular torsion, universally accepted normative limits have not been established. DFA measurements may be influenced by both technical and anatomical factors, including image acquisition technique, head positioning and fixation during photography, as well as individual variability in optic disc and foveal anatomy. Differences between imaging modalities may further affect measurements, emphasizing the importance of standardized acquisition and analysis protocols. Reported mean DFA values vary across studies, most commonly ranging from approximately 5°–7.5°, depending on the population studied, imaging modality, measurement technique, and definition of anatomical landmarks (Bixenman and von Noorden, 1982; Williams and Wilkinson, 1992; Kanamori et al., 2012; Hirasawa et al., 2015; Shin et al., 2013; Simiera et al., 2021; Lengwiler et al., 2018; Le Jeune et al., 2018). This variability should be considered when interpreting individual measurements, particularly in borderline cases. Therefore, DFA is best interpreted in the context of published normative data, interocular asymmetry, clinical findings, and, when available, longitudinal or pre- and postoperative changes. Mean DFA values reported in the literature are summarized in Table 3.

**TABLE 3:** Mean disc–foveal angle (DFA) values reported in the literature.

Publication	Mean DFA value
Bixenman and von Noorden (1982)	7.25 ± 2.57°
Williams and Wilkinson (1992)	6.11 ± 3.3°
Kanamori et al. (2012)	7.4 ± 3.8°
Hirasawa et al. (2015)	7.2 ± 3.4°
Shin et al. (2013)	5.13 ± 2.79°
Simiera and Loba (2017)	6.39 ± 2.72°
Lengwiler et al. (2018)	6.6 ± 2.8°
Le Jeune et al. (2018)	6.89 ± 4.41°

## Translational implications for surgical and medical treatment

Quantitative assessment of ocular cyclotorsion has important translational implications for both surgical planning and medical management across a broad spectrum of ophthalmic and neuro-ophthalmic disorders. In strabismus, objective measurement of torsion complements horizontal and vertical deviation assessments by revealing underlying oblique muscle dysfunction, pulley abnormalities, or vestibular imbalance that may not be evident on routine ocular alignment testing. Preoperative torsional evaluation assists in selecting the most appropriate surgical strategy, determining the extent of oblique muscle weakening or strengthening procedures, and identifying patients who may benefit from certain techniques. Postoperative assessment of cyclotorsion provides an objective marker of surgical efficacy and may explain persistent diplopia or residual binocular dysfunction despite satisfactory motor alignment.

## Pathological cyclotorsion

Cyclotropia, or manifest cyclodeviation, refers to abnormal torsional positioning of the eye relative to the vertical axis, whereas cyclophoria represents a latent torsional deviation that is usually controlled by fusional mechanisms (Wick and Ryan, 1982). Cyclodeviation may result from imbalance or dysfunction of the extraocular muscles responsible for torsional eye movements: the superior oblique and superior rectus muscles contribute to intorsion, while the inferior oblique and inferior rectus muscles contribute to extorsion (Rucker and Tomsak, 2005; Horn and Leigh, 2011). Compensation occurs predominantly through sensory cyclofusion, which can correct several degrees of torsional disparity, whereas the motor component, mediated by cyclovergence, provides a smaller corrective contribution (Guyton, 1988; Herzau and Joos-Kratsch, 1984). Therefore, the clinical manifestation of cyclodeviation depends not only on the magnitude of anatomical torsion, but also on the patient’s capacity for sensory and motor fusion.

## Trochlear nerve palsy

Trochlear nerve palsy is one of the most common neurogenic causes of cyclovertical strabismus and results from dysfunction of the superior oblique muscle (Chang et al., 2017; Dosunmu et al., 2018). Because as mentioned before the superior oblique acts primarily as an intortor, as well as a depressor and abductor when the eye is adducted, its weakness typically produces hypertropia and excyclotorsion of the affected eye, with limitation of depression in adduction. These characteristic ocular motility findings are illustrated in Figure 4. The vertical deviation is often incomitant and typically increases in contralateral gaze and with ipsilateral head tilt, which forms the basis of the Bielschowsky head tilt test. Patients may present with vertical or torsional diplopia, often worse in downgaze and near tasks such as reading or descending stairs, and may adopt a compensatory head tilt away from the affected side to reduce symptoms (Bixenman, 1981). Congenital cases may remain partially compensated for many years because of large vertical fusional amplitudes and may be associated with long-standing facial asymmetry or chronic head posture, whereas acquired trochlear nerve palsy more commonly produces acute diplopia and symptomatic torsional misalignment (Choi et al., 2024). Assessment of ocular torsion is therefore important in both diagnosis and surgical planning, particularly when differentiating trochlear nerve palsy from other causes of cyclovertical deviation such as skew deviation, restrictive strabismus, or pulley disorders.

**FIGURE 4:**
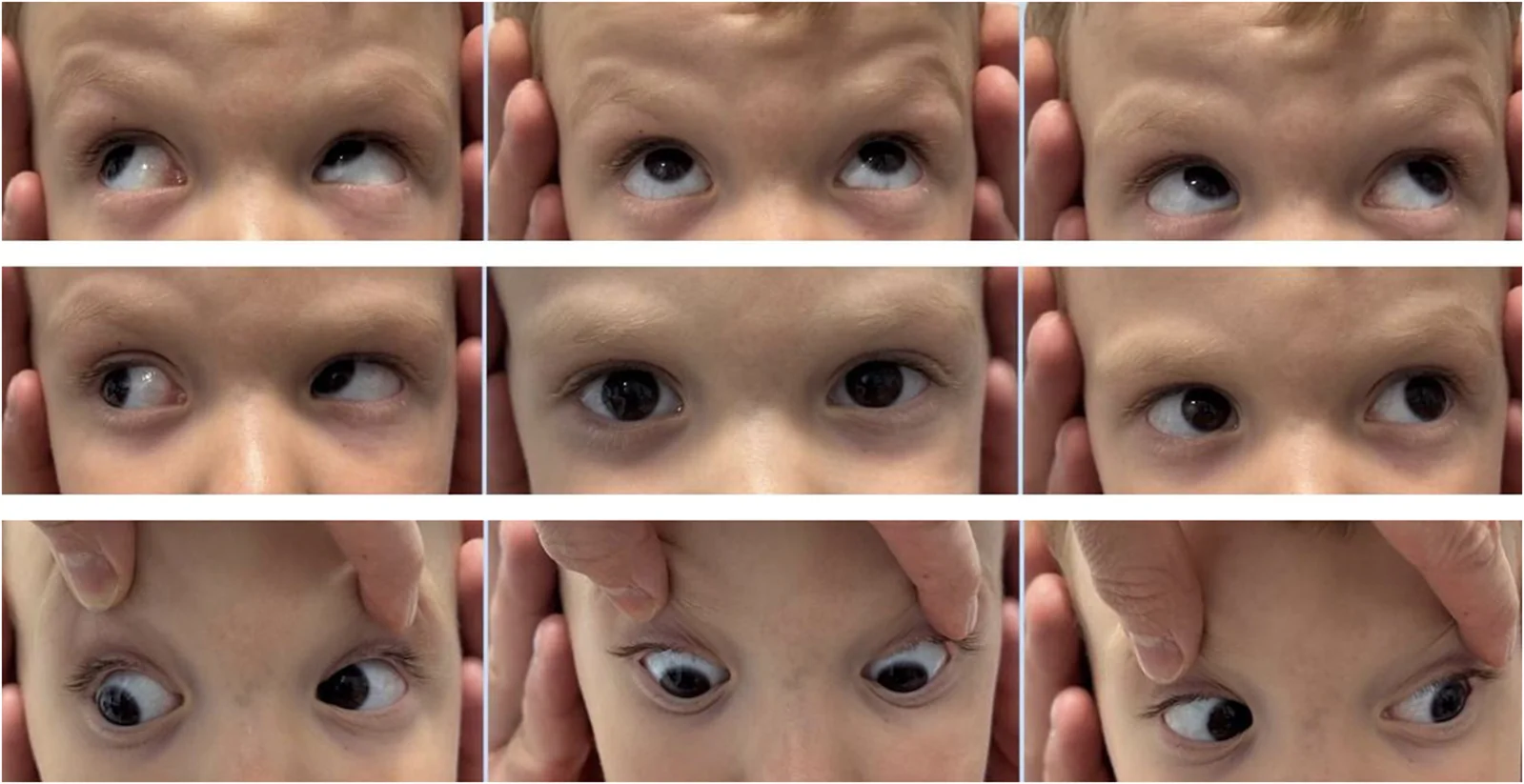
Ocular motility examination in a patient with congenital right trochlear nerve palsy with over-elevation of the right eye in adduction and reduced depression of the right eye in adduction.

## Skew deviation and ocular tilt reaction

Lesions involving the brainstem, cerebellum, or vestibular pathways may disturb ocular torsion by disrupting vestibulo-ocular mechanisms responsible for maintaining alignment in the roll plane (Dieterich and Brandt, 1993). Damage to otolithic projections extending from the peripheral vestibular organs through the vestibular nuclei, brainstem, and cerebellum may lead to an imbalance of vestibular tone, resulting in binocular ocular torsion, skew deviation, head tilt, and altered perception of the subjective visual vertical (Choi et al., 2024). Skew deviation is thought to arise from a unilateral lesion affecting the central otolithic pathways that ascend from the medulla to the mesencephalon (Brandt and Dieterich, 1994), most commonly through reduced pathway activity and, less frequently, through abnormal stimulation. When these findings occur together, they constitute the ocular tilt reaction, a characteristic manifestation of disturbed otolith-ocular control. In contrast to trochlear nerve palsy, in which torsion is usually monocular and related to superior oblique weakness, torsion caused by brainstem, cerebellar, or vestibular lesions is typically binocular and often associated with other neurological or vestibular signs, such as nystagmus, vertigo, ataxia, or gaze-holding abnormalities (Wong, 2010). Recognition of this pattern is clinically important, because acquired cyclotorsion with skew deviation or ocular tilt reaction may indicate central vestibular, brainstem, or cerebellar pathology and should prompt appropriate neuro-ophthalmic and neurological evaluation.

## Pulley disorders

Structural abnormalities of the extraocular muscle pulley system are recognized as an important mechanical cause of cyclotorsion and incomitant strabismus. Rectus muscle pulleys are fibroelastic connective tissue sleeves that constrain extraocular muscle paths and act as functional mechanical origins, thereby determining the direction of muscle force vectors and influencing ocular rotational kinematics (Demer et al., 1995; Clark et al., 1997; Demer, 2004). Consequently, congenital pulley heterotopy, acquired pulley instability, or rotational displacement of the pulley array may alter rectus muscle pulling directions and produce cyclovertical or pattern strabismus, sometimes mimicking primary oblique muscle dysfunction (Clark et al., 1998). Craniosynostosis syndromes, particularly Crouzon, Apert, and Pfeiffer syndromes, provide an important clinical example of this mechanism, as abnormal cranio-orbital development may be associated with excyclorotation of the orbits, rectus muscle cone, and rectus pulley array. This can result in V-pattern strabismus, apparent inferior oblique overaction, over-elevation in adduction, and fundus excyclotorsion (Tan et al., 2005; Weiss et al., 2014; Dagi et al., 2017). Imaging studies in Crouzon syndrome have shown that extorsion of the rectus muscle pulleys can reproduce the observed V-pattern deviation, supporting a primarily mechanical mechanism (Weiss et al., 2014). Similarly, quantitative studies in syndromic craniosynostosis have demonstrated an association between the severity of V-pattern strabismus and the degree of rectus muscle excyclorotation (Dagi et al., 2017). These observations have substantially changed the understanding of selected forms of cyclovertical strabismus, shifting the interpretation from isolated abnormalities of extraocular muscle contractility toward disturbances of orbital biomechanics. Pulley-related abnormalities may be congenital, as in pulley heterotopy and craniosynostosis, or acquired, as in age-related connective tissue degeneration, high myopic globe elongation, or inflammatory and fibrotic orbital disease.

## Inferior oblique overaction

Inferior oblique overaction (IOOA) is frequently associated with V-pattern strabismus and represents an important contributor to both vertical incomitance and ocular torsion. V-pattern strabismus along with A-pattern strabismus are among the most common alphabetic patterns (von Noorden and Campos, 2002), occurring in 11%–50% of patients with strabismus (Zhu et al., 2022). In V-pattern strabismus, the horizontal deviation is more convergent, or less divergent, in downgaze than in upgaze (Ghasia and Shaikh, 2013), with clinically significant cases typically defined by a difference of more than 15 prism diopters (△) between upgaze and downgaze. Although early theories attributed V-pattern strabismus mainly to dysfunction of the horizontal and vertical rectus muscles (Knapp, 1959), current concepts more often emphasize the role of the oblique muscles, particularly the inferior oblique. Because the inferior oblique muscle has vertical, horizontal, and torsional actions, its overaction may produce excessive elevation in adduction, contribute to the V-pattern deviation, and induce excyclotorsion. Kushner proposed a theory in which primary oblique muscle overaction induces torsion of the globe, thereby altering the spatial position of the rectus muscle insertions. According to this concept, torsion may cause vertical displacement of the horizontal rectus insertions and horizontal displacement of the vertical rectus insertions, modifying the effective force vectors acting on the globe. As a result, the horizontal recti may acquire secondary elevating or depressing actions, whereas the vertical recti may exert increased abducting or adducting effects. These changes may amplify the A- or V-pattern deviation associated with oblique muscle dysfunction, suggesting that alphabetic patterns arise from a complex interaction among all extraocular muscles rather than from isolated dysfunction of the oblique muscles alone (Kushner, 1985). Later in the literature, the pathogenesis of A- and V-pattern strabismus was increasingly regarded as multifactorial, involving both mechanical and neural mechanisms. Mechanical factors may involve abnormalities of the extraocular muscle pulleys (Demer et al., 2000; Demer et al., 2003), whereas neural mechanisms may include loss of fusion with abnormal torsion (Miller and Guyton, 1994), disrupted supranuclear control (Das et al., 2005), and vestibular hypofunction (Donahue and Itharat, 2010). Most V-pattern deviations are congenital or develop early in life, allowing sensory adaptations such as suppression or anomalous retinal correspondence to reduce subjective awareness of torsion. Despite these sensory adaptations, objective excyclotorsion in patients with V-pattern strabismus and inferior oblique overaction may be detected clinically and documented on fundus photographs, as shown in Figure 5, which presents pre- and postoperative images and Cyclocheck®-based assessment of the disc–foveal angle (DFA) used to quantify excyclotorsion.

**FIGURE 5:**
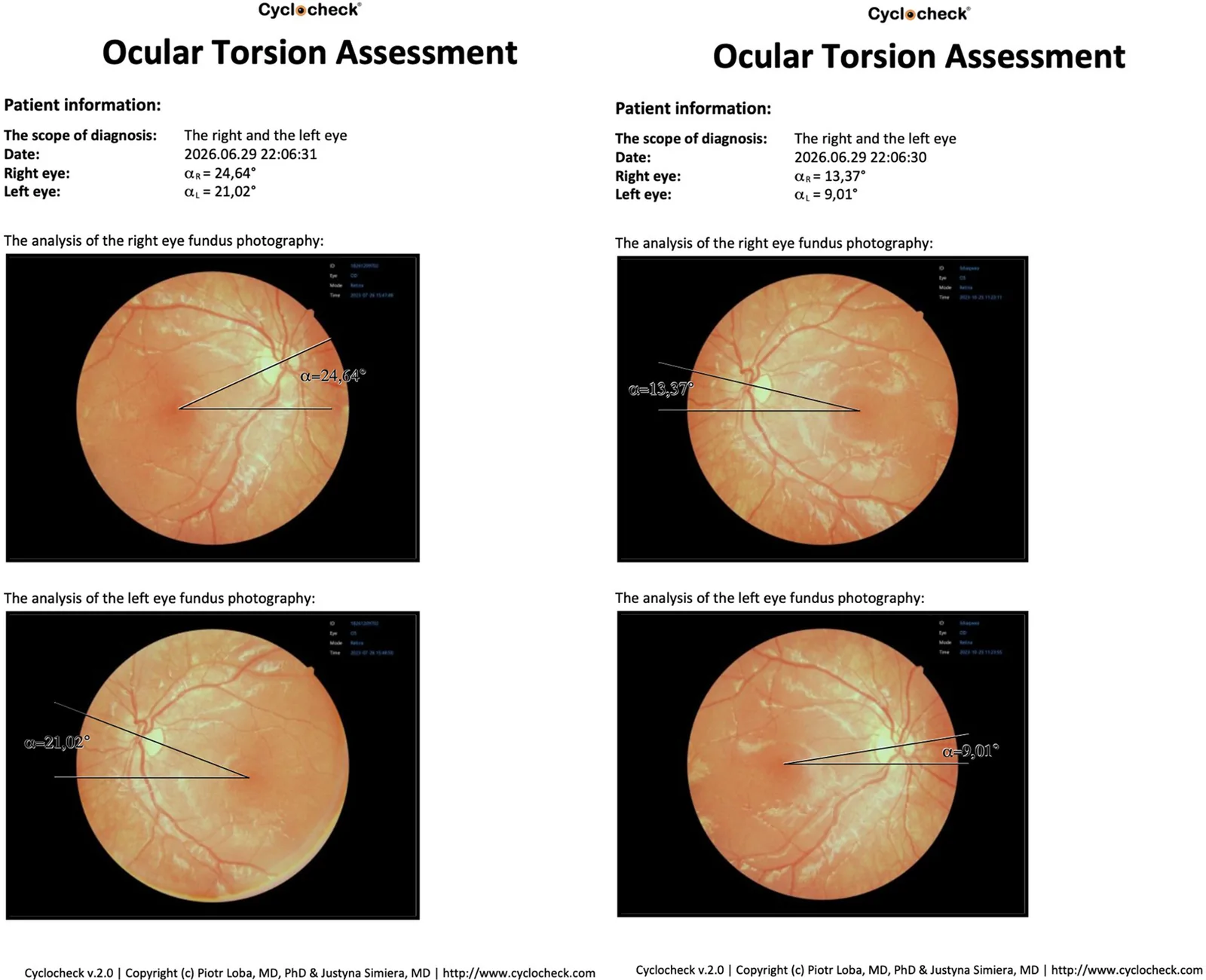
Pre- and postoperative fundus photographs and objective cyclotorsion assessment using the Cyclocheck® application. The disc–foveal angle (DFA) was measured to quantify excyclotorsion in a patient with V-pattern strabismus and inferior oblique overaction. Author’s own material.

## Thyroid eye disease

Thyroid eye disease (TED), also known as Graves’ orbitopathy, is an autoimmune inflammatory disorder of the orbit most commonly associated with Graves’ disease. It is characterized by immune-mediated inflammation and remodeling of the extraocular muscles, orbital connective tissue, and adipose tissue, leading to enlargement and fibrosis of orbital structures (Bartalena et al., 2021; Yoon and Kikkawa, 2022). Clinically, TED may present with eyelid retraction, proptosis, exposure-related ocular surface disease, restrictive strabismus, and diplopia. Involvement of the extraocular muscles, particularly the inferior and medial rectus muscles, can alter ocular motility and contribute to vertical, horizontal, and less commonly, torsional deviations (Akbari and Mirmohammadsadeghi, 2020).

Torsional misalignment in TED may cause disabling diplopia, abnormal head posture, and significant visual discomfort; therefore, evaluation of ocular torsion represents an important component of the diagnostic and surgical assessment of TED-related strabismus, alongside ocular duction testing, forced duction testing, and orbital imaging. Quantification of torsional deviation may provide clinically relevant information regarding the pattern and severity of extraocular muscle involvement and may assist in both surgical planning and postoperative outcome assessment. Tran et al. (2023) evaluated 40 patients with TED-related vertical strabismus and found a torsional component in all cases, with most patients exhibiting excyclotorsion associated with restricted elevation, and a smaller subgroup demonstrating incyclotorsion linked to limited depression. Vertical rectus muscle surgery significantly reduced ocular torsion without the need for additional oblique muscle procedures and substantially improved binocular single vision, with the magnitude of postoperative torsional correction related to the degree of preoperative torsion. These findings suggest that cyclotorsion is a common feature of TED-associated strabismus and that vertical rectus muscle surgery alone may often be sufficient to address both vertical and torsional misalignment. However, torsional deviations in TED may also arise from oblique muscle involvement. Holmes et al. (2012) described masked superior oblique tightness in patients with TED, which may become apparent after inferior rectus recession and lead to postoperative A-pattern exotropia with intorsion. Accordingly, careful assessment of ocular torsion and superior oblique restriction may be particularly important in patients with inferior rectus involvement to reduce the risk of postoperative torsional diplopia.

## Heavy eye syndrome

High myopia represents a clinically important form of myopia associated with increased axial elongation and structural changes of the posterior segment. Although myopia has traditionally been classified according to spherical equivalent refractive error, with high myopia defined as greater than −6.00 D, refractive error alone does not fully reflect the risk of ocular complications. For this reason, current approaches increasingly emphasize axial length, with values of approximately 26.0 mm or greater considered a more reliable marker of long-term risk, including retinal detachment, myopic macular degeneration, and glaucoma. In high myopia, progressive stretching and thinning of the sclera, choroid, and retina may contribute to clinically significant anatomical and functional consequences (Tideman et al., 2016; Du et al., 2024; Holden et al., 2016).

Heavy eye syndrome (HES), also known as myopic strabismus fixus, is an acquired form of restrictive strabismus associated with high axial myopia. (Sturm et al., 2008). Excessive elongation of the globe results in superotemporal prolapse of the posterior pole within the orbit, altering the normal anatomical relationship between the globe and the extraocular muscle cone (Hennein and Robbins, 2021; Pineles, 2022). This displacement leads to secondary changes in the paths of the horizontal and vertical rectus muscles, most commonly inferior displacement of the lateral rectus and nasal displacement of the superior rectus, thereby contributing to progressive esotropia and hypotropia. Clinically, it typically presents with progressive esotropia and hypotropia, often accompanied by limitation of abduction and elevation (Tan and Demer, 2015; Hayashi et al., 1999).

Although cyclotorsion has not been extensively studied as a primary outcome in heavy eye syndrome, the marked displacement of the globe and extraocular muscle paths in highly myopic strabismus may theoretically alter torsional forces. Therefore, assessment of objective cyclotorsion may provide an additional anatomical and functional parameter in patients with heavy eye syndrome, particularly when evaluating preoperative status and postoperative changes.

## Sagging eye syndrome

Sagging eye syndrome (SES) is an acquired, age-related form of strabismus caused by degeneration of the orbital connective tissues that support the extraocular muscle pulley system. It is increasingly recognized as one of the most common causes of acquired non-paralytic binocular diplopia in older adults, with its prevalence rising markedly with age and a slight female predominance of approximately 60% (Goseki, 2021). More recently, Yehezkeli et al. (2026) reported that SES is associated with advanced age at symptom onset in both sexes and, in women, with lower body mass index and osteopenia. Patients with SES may show characteristic external adnexal features, including baggy eyelids, deep superior sulcus deformity, and aponeurotic ptosis (Chaudhuri and Demer, 2013). The most characteristic anatomical change is laxity or rupture of the lateral rectus–superior rectus band, resulting in inferolateral displacement of the lateral rectus pulley and altered rectus muscle force vectors. These changes may produce distance esotropia, small-angle vertical deviation, or cyclovertical strabismus, particularly in older adults without neurological disease. When pulley displacement is asymmetric between the two eyes, the more inferiorly displaced lateral rectus pulley may induce hypotropia and may be associated with excyclotorsion (Chaudhuri and Demer, 2013). Therefore, assessment of ocular torsion can be useful in patients with SES presenting with vertical or cyclovertical diplopia. Recognition of SES is important because its clinical presentation may mimic cranial nerve palsy, while its pathogenesis is primarily mechanical and related to orbital connective tissue involution.

## Discussion

Cyclotorsion is an important, though often underestimated, component of ocular motility that plays a key role in the proper functioning of the visual system, binocular vision, and spatial orientation. Physiologically, torsional eye movements contribute to stabilization of retinal image orientation during changes in head and body position, while also supporting binocular fusion and accurate perception of verticality. The ocular counter-roll reflex represents one of the major physiological mechanisms responsible for torsional compensation during head tilt and depends on the integration of vestibular, visual, brainstem, and cerebellar inputs (Reschke et al., 2018; Kheradmand and Zee, 2011; Brodsky, 2022). These movements are generated mainly by the coordinated action of the oblique muscles, with additional contribution from the vertical rectus muscles, and their balance is essential for maintaining stable ocular alignment (Horn and Leigh, 2011; Rucker and Tomsak, 2005). The assessment of cyclotorsion is particularly significant in the diagnosis and treatment of vertical and oblique strabismus, where even minor torsional deviations may influence symptoms, treatment planning, and postoperative outcomes. Disturbances of torsional alignment may manifest as torsional diplopia, abnormal head posture, impaired fusion, or nonspecific visual discomfort; however, the relationship between anatomical torsion and subjective symptoms is not always direct. Sensory adaptation, suppression, and cyclofusion may reduce or mask symptoms, particularly in congenital or long-standing deviations (Guyton, 1988; Herzau and Joos-Kratsch, 1984; Kushner and Hariharan, 2009). For this reason, subjective tests such as the double Maddox rod test, synoptophore, and Harms tangent screen remain useful for evaluating perceived torsion, but should be interpreted together with objective anatomical assessment (Klainguti et al., 1992; Simons et al., 1994; Llorente-La Orden et al., 2022). Objective evaluation of cyclotorsion, most commonly based on fundus imaging and measurement of the disc–foveal angle, provides reproducible information about the anatomical position of the posterior pole. This is particularly valuable because objective torsion may be present even when subjective complaints are mild or absent. The disc–foveal angle has therefore become an important parameter in both clinical assessment and research, although published normative values vary depending on population, imaging technique, and measurement methodology (Bixenman and von Noorden, 1982; Jonas et al., 2015; Lengwiler et al., 2018; Le Jeune et al., 2018). Standardized digital tools, including Cyclocheck®, may further improve the reliability and repeatability of cyclotorsion measurements and facilitate comparison between preoperative and postoperative findings (Simiera and Loba, 2017; Simiera et al., 2021). Pathological cyclotorsion may result from a wide range of neurogenic, vestibular, restrictive, mechanical, and orbital mechanisms. It is particularly relevant in trochlear nerve palsy, where superior oblique dysfunction typically produces excyclotorsion and vertical diplopia (Bixenman, 1981; Chang et al., 2017; Choi et al., 2024). Cyclotorsion may also occur in skew deviation and ocular tilt reaction due to disruption of otolith-ocular pathways, often indicating brainstem, cerebellar, or vestibular involvement (Dieterich and Brandt, 1993; Brandt and Dieterich, 1994; Wong, 2010). In mechanical forms of strabismus, such as pulley disorders, craniosynostosis-related pattern strabismus, sagging eye syndrome, and heavy eye syndrome, torsional abnormalities may reflect altered extraocular muscle paths or orbital connective tissue changes rather than isolated muscle weakness (Demer et al., 1995; Clark et al., 1998; Chaudhuri and Demer, 2013; Tan and Demer, 2015). Similarly, in thyroid eye disease, restrictive involvement of the extraocular muscles may produce clinically relevant torsional components that influence both surgical strategy and postoperative binocular function (Holmes et al., 2012; Tran et al., 2023).

Overall, cyclotorsion should be regarded as both a functional and structural biomarker of ocular motor balance. Its clinical utility is best established in conditions such as trochlear nerve palsy, skew deviation, and pattern strabismus associated with oblique muscle dysfunction, where torsional assessment can contribute to diagnosis and treatment planning. In other disorders, including heavy eye and sagging eye syndromes, its role as a biomarker remains more exploratory and requires further validation. Systematic evaluation of cyclotorsion may enhance the understanding of complex strabismus mechanisms, improve differentiation between paralytic, restrictive, vestibular, and mechanical etiologies, and support more individualized treatment strategies. Incorporating cyclotorsion measurement into routine ophthalmological and neuro-ophthalmological assessment may therefore contribute to more precise diagnosis, better surgical planning, and more reliable evaluation of treatment outcomes in patients with torsional misalignment.

## Future perspectives and concluding remarks

Advances in digital fundus photography, optical coherence tomography (OCT), scanning laser ophthalmoscopy, and video-oculography have enabled increasingly accurate, reproducible, and non-invasive quantification of cyclotorsion, facilitating longitudinal follow-up and objective outcome assessment. As imaging technologies continue to evolve, cyclotorsion has the potential to serve not only as a diagnostic marker but also as a quantitative biomarker for individualized treatment planning, prognostication, and evaluation of therapeutic interventions in ocular and neurological disease. Future research should focus on standardizing cyclotorsion measurement across imaging modalities and establishing large normative databases that account for age, refractive status, and ocular anatomy.

Artificial intelligence and deep-learning–based retinal image analysis may further improve objective torsion assessment. Automated algorithms have already demonstrated the feasibility of localizing the optic disc and fovea, calculating the disc–fovea angle from fundus photographs, and detecting static intorsion and extorsion (Zheng et al., 2022; Wang et al., 2023). More recent automated approaches may further improve the speed and reproducibility of DFA assessment (Hirota et al., 2026). In parallel, OCT-based measurements of ocular torsion have shown good agreement with conventional fundus photography, creating opportunities for integration of complementary structural information from both modalities (Yamadera et al., 2020; Liu et al., 2025). Multimodal artificial intelligence combining fundus photography and OCT has already shown promising results in broader retinal image analysis (Sükei et al., 2024) and may provide a framework for future cyclotorsion assessment. Emerging approaches combining fundus photography, OCT, high-resolution eye tracking, wearable video-oculography, and, where appropriate, orbital or neuroimaging data may provide a more comprehensive assessment of both static and dynamic cyclotorsion under physiological and pathological conditions. Such approaches could improve understanding of disease mechanisms while providing sensitive biomarkers for early diagnosis, monitoring disease progression, predicting treatment response, and optimizing individualized surgical and medical management.

However, current automated approaches remain limited by relatively small or single-center datasets, differences in imaging protocols and devices, and limited external validation. Prospective multicenter studies using standardized acquisition and analysis protocols across diverse populations and clinical conditions will therefore be essential to validate AI-based and multimodal approaches and to establish cyclotorsion as a robust structural and functional biomarker in ophthalmology.
